# A portion of expanded granular lymphocytes cause pure white cell aplasia?

**DOI:** 10.1007/s00277-018-3342-5

**Published:** 2018-05-02

**Authors:** Yasushi Isobe, Yu Uemura, Akiko Uchida, Ikuo Miura

**Affiliations:** Division of Hematology & Oncology, Department of Internal Medicine, St. Marianna University School of Medicine, 2-16-1 Sugao, Miyamae-ku, Kawasaki, Kanagawa 216-8511 Japan

Dear Editor,

Pure white cell aplasia (PWCA) is a rare disorder characterized by selective hematopoietic failure of both granulocytic and monocytic lineages [1]. Herein we report a case with T cell granular lymphocyte (GL)-proliferative disorders (T-GLPD)-associated PWCA, which acquired prolonged remission from PWCA in spite of that GL counts had no apparent change before and after receiving alemtuzumab therapy.

**Fig. 1:**
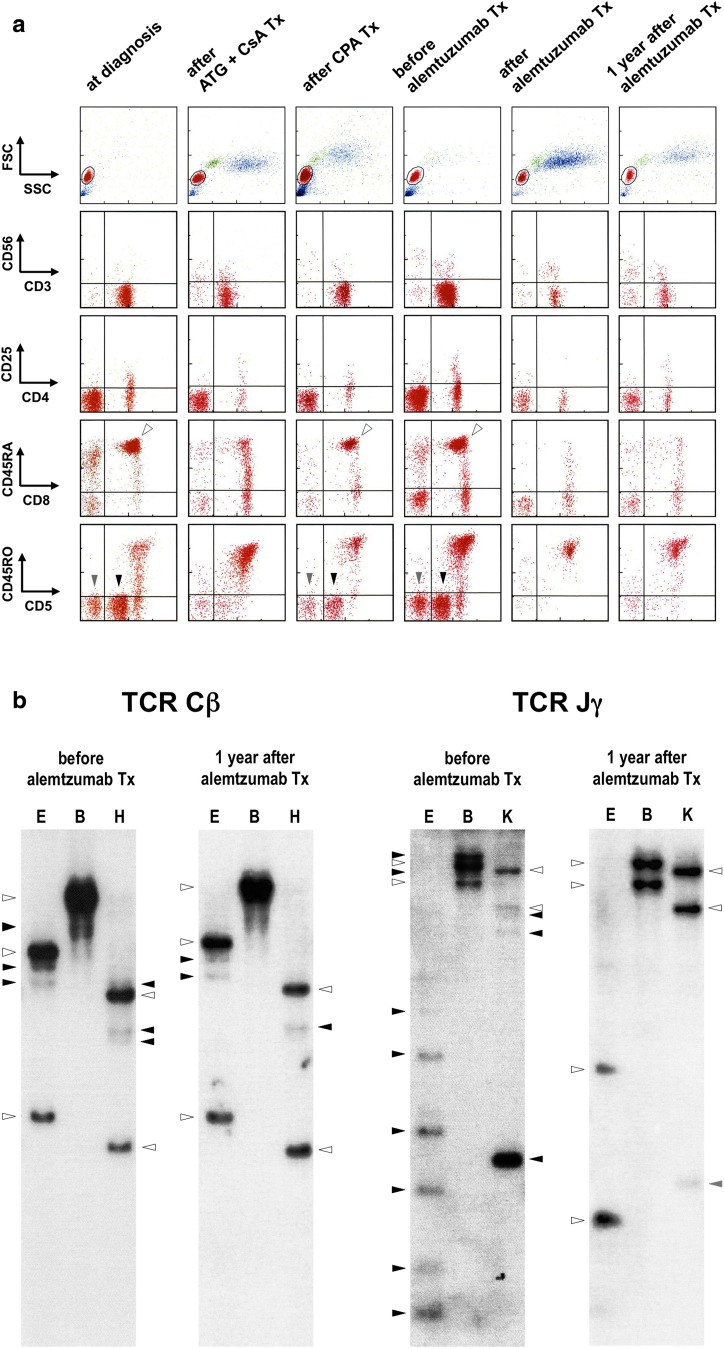
**a** Flow cytometry showed 96.5% of the peripheral blood (PB) lymphocytes (red cell fractions) were CD3^+^ T cells comprising of CD4^+^ (17.7%), CD8^+^ (71.1%), and CD4^−^CD8^−^ cells (7.7%) at diagnosis. In the CD45RA^+^CD45RO^−^CD8^+^ T cell fraction including effector cytotoxic T lymphocytes (shown by open arrowheads), there were unique populations of CD5^−^ and CD5^low^ T cells (shown by gray and black arrowheads, respectively). Although they failed to be eradicated by immunosuppressive therapies (Txs) with anti-thymocyte globulin (ATG) plus cyclosporine A (CsA) and oral cyclophosphamide (CPA), these abnormal populations disappeared after receiving alemtuzumab Tx. **b** Southern blot analyses of T cell receptor (TCR) gene rearrangements confirmed the clonal T cell proliferation in the PB before receiving alemtuzumab Tx. Each genomic DNA was extracted from the PB mononuclear cells, electrophoresed after digestion with *Eco*RI (E), *Bam*HI (B), and *Hin*dIII (H) or *Kpn*I (K), transferred onto a nylon membrane, hybridized with TCR Cβ or Jy probes, and detected using chemiluminescence. Germline and rearranged bands are shown by open and black arrowheads, respectively. The pseudogene is shown by a gray arrowhead. Although the clonal bands of TCR Jy gene rearrangement disappeared, the Cβ gene bands unexpectedly remained to be detected even 1 year after receiving alemtuzumab Tx

A 66-year-old female was referred to our hospital because of leukopenia. Neither lymphadenopathy nor splenomegaly was found by physical examination or computed tomography. The white blood cell count was 1.5 × 10^9^/L (58% GLs, 41% other lymphocytes, and 1% basophils). A bone marrow examination revealed slightly hypocellular marrow with maturation arrest in granulocytic and monocytic lineages. The patient was diagnosed with PWCA. In the peripheral blood (PB), the unique CD5^−^ and CD5^low^ T cell populations were clearly detected in the CD45RA^+^CD45RO^−^CD8^+^ fraction including effector cytotoxic T lymphocytes (CTLs) (Fig. 1a). Although the GL count has never exceeded 1.1 × 10^9^/L during the clinical course, *STAT3* D661Y allele was detected [2]. Southern blot analyses of T cell receptor (TCR) gene rearrangements confirmed that most GLs were clonally expanded (Fig. 1b). According to the revised World Health Organization classification, persistent (> 6 months) clonal expansion of CTL-type GLs in the PB (at least >2 × 10^9^/L) is defined as T cell large GL leukemia (T-LGLL), which has been recognized as a neoplasm [3]. Therefore, we regarded this case failing to meet the diagnostic criteria for T-LGLL as T-GLPD and considered that the expanded GLs may cause the PWCA [4, 5].

The patient initially received immunosuppressive therapy including cyclosporine A and rabbit anti-thymocyte globulin. Absolute neutrophil count (ANC) was elevated to 1.49 × 10^9^/L 3 months after receiving the therapy. Shortly thereafter, the neutrophil and monocyte counts were gradually decreased below 0.5 × 10^9^/L. CD5^−^ and CD5^low^ CTLs reappeared, suggesting the recurrence of PWCA (Fig. 1a). The patient next received oral cyclophosphamide, while the pathogenetic cell populations failed to be eradicated (Fig. 1a). She further received low-dose alemtuzumab therapy. After a 3 mg initial dose, alemtuzumab was administered intravenously at 10 mg once a week, resulting in a total dose of 163 mg. Again, rapid recovery of the ANC was observed concomitantly with the disappearance of CD5^−^ and CD5^low^ CTLs (Fig. 1a). Although Southern blot analysis of TCR Jγ gene showed no clonal bands, the Cβ gene rearranged bands continued to be detected even 1 year after receiving the alemtuzumab therapy (Fig. 1b). The patient remained well without the recurrence of PWCA in spite of that GL fractions accounted for around 30% of lymphocytes.

The prolonged resolution of PWCA with disappearance of CD5^−^ and CD5^low^ effector CTLs suggests the cytotoxicity-related etiology. Even though all clones failed to be eradicated, cytopenia was resolved when the pathogenetic CTLs disappeared after the alemtuzumab therapy. Heterogeneity in clonal expanded GLs suggests that the present case has a reactive condition rather than leukemia-like neoplasms showing homogeneous cell populations. It may be appropriate to consider T-GLPD and the true neoplastic leukemia separately in T-LGLL.
